# Clinical, cellular, microscopic, and ultrastructural studies of a case of fibrogenesis imperfecta ossium

**DOI:** 10.1038/boneres.2016.57

**Published:** 2017-03-14

**Authors:** Melissa L Barron, Mark S Rybchyn, Sutharshani Ramesh, Rebecca S Mason, S Fiona Bonar, Paul Stalley, Sundeep Khosla, Bernie Hudson, Christopher Arthur, Edward Kim, Roderick J Clifton-Bligh, Phillip B Clifton-Bligh

**Affiliations:** 1 Department of Physiology, School of Medical Sciences, Bosch Institute, University of Sydney, Sydney 2006, New South Wales, Australia; 2 Douglas HanlyMoir Pathology, Macquarie Park 2113, New South Wales, Australia; 3 Department of Orthopaedics, Royal Prince Alfred Hospital, Camperdown 2050, New South Wales, Australia; 4 Department of Endocrinology, Mayo Clinic, Rochester 55905, MN, USA; 5 Department of Microbiology, Royal North Shore Hospital, St Leonards 2065, New South Wales, Australia; 6 Department of Haematology, Royal North Shore Hospital, St Leonards 2065, New South Wales, Australia; 7 Department of Endocrinology, Royal North Shore Hospital, St Leonards 2065, New South Wales, Australia; 8 Faculty of Medicine, University of Sydney, Sydney 2006, New South Wales, Australia

## Abstract

Fibrogenesis imperfecta ossium is a rare disorder of bone usually characterized by marked osteopenia and associated with variable osteoporosis and osteosclerosis, changing over time. Histological examination shows that newly formed collagen is abnormal, lacking birefringence when examined by polarized light. The case presented demonstrates these features and, in addition, a previously undocumented finding of a persistent marked reduction of the serum C3 and C4. Osteoblasts established in culture from a bone biopsy showed abnormal morphology on electron microscopy and increased proliferation when cultured with benzoylbenzoyl-ATP and 1,25-dihydroxyvitamin D, contrasting with findings in normal osteoblasts in culture. A gene microarray study showed marked upregulation of the messenger RNA (mRNA) for G-protein-coupled receptor 128 (GPR 128), an orphan receptor of unknown function and also of osteoprotegerin in the patient’s osteoblasts in culture. When normal osteoblasts were cultured with the patient’s serum, there was marked upregulation of the mRNA for aquaporin 1. A single pathogenetic factor to account for the features of this disorder has not been defined, but the unique findings described here may facilitate more definitive investigation of the abnormal bone cell function.

## Introduction

The case is presented as an example of a rare bone disorder, fibrogenesis ossium imperfecta. In addition to a description of the clinical features at presentation and subsequently, hitherto unreported blood test findings are described and an analysis of the function of osteoblasts cultured from affected bone is given. A gene microarray was also performed on the cultured osteoblasts looking at the expression of genes that might be involved in the bony abnormalities described.

The patient, a male student, aged 17 years, was first seen on 14 August 2002 with a painful right knee incurred while playing soccer. A magnetic resonance imaging of the right knee showed an anterior cruciate ligament tear and adjacent bone bruising but no other abnormality in the distal femur or proximal tibia. The ligament was surgically repaired and he recovered well from this injury. In June 2006, he had a febrile illness with a sore throat treated with amoxicillin. One month later, he developed polyarthralgia involving the knees, ankles, elbows, and wrists. There was no early morning stiffness or swelling of the joints. Over the next 2 years, he had several more episodes of sore throat treated with injections of procaine penicillin. During this time, the knee and ankle pain became more severe and constant made worse by drinking alcohol and was less after receiving penicillin. The last course of penicillin, given orally, was in May 2008. On the 19 July 2007, he was seen by a rheumatologist. An examination of his joints was normal. A serum C reactive protein (CRP) was 35 mg·L^−1^, elevated. Tests for rheumatoid factor and for antinuclear antibody were negative. He was thought to have a post-streptococcal syndrome. He was seen by a microbiologist on 13 September 2007. At this time, the serum C3 and C4 were found to be markedly reduced. No evidence for a persisting infection was found.

On examination at presentation to us on 5 April 2008, his blood pressure was 100/70 mmHg. Examination of the heart, neck, lungs, and abdomen was unremarkable and the tendon reflexes were normal. There was no joint swelling, tenderness, or limitation of movement and muscle power was normal. There was loss of lordosis of the lumbar spine. There was no family history of bone or joint disorder. His father had been treated successfully for chronic myeloid leukemia. Investigations carried out in 2007 and 2008 are shown in Table 1. The tests focused on the possibility that an infection might be relevant to the joint disorder or that a generalized bone disorder might be present. The markedly reduced serum C3 and C4 was confirmed on repeated testing. There was a circulating IgG kappa monoclonal protein that has shown no tendency to rise progressively over the subsequent several years of testing.

**Figure 1:**
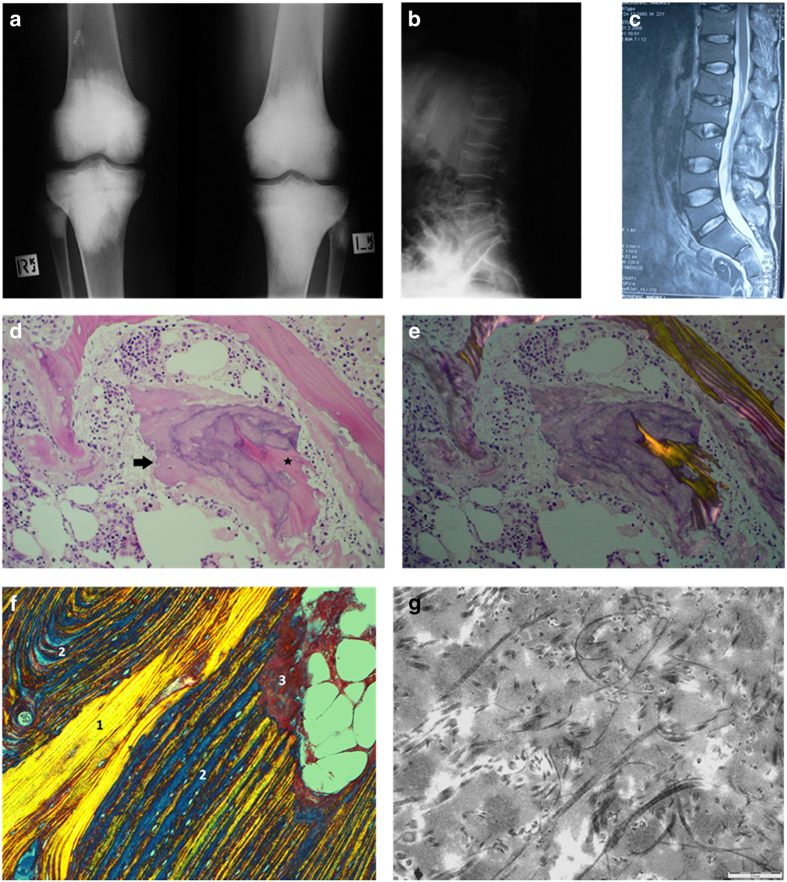
Radiograph of the knees (**a**) shows marked bilateral symmetrical homogeneous sclerosis involving the epiphyses and metaphyses, involving all bones related to the joint. Radiograph and T2-weighted MR image (**b** and **c**) shows central collapse of the vertebral end plates associated with osteoporosis. In the iliac crest bone marrow trephine, variably sized bone trabelculae are noted with intervening haemopoeitic marrow (**d**). In thicker trabeculae, wide irregular osteoid seams with calcification are noted (arrowed). Normal slender trabeculae of lamellar bone are presented focally. The intervening immature osteoid has irregular zones of calcification stained in blue. Centrally, a residual spicule of more dense pink normal lamellar bone (star) is bordered by zones of immature pink osteoid with irregular poorly defined zones of calcification, the calcification predominating where the osteoid abuts the bone trabeculum and diminished at the perimeter (arrowed). On polarization microscopy of the same area (**e**), the original host lamellar bone with generous quantities of collagen birefringence is minimal. In the femur biopsy, a Masson trichrome stain examined under polarized light (**f**) shows original host bone with cohesive and generous collagen content (zone 1). This is bordered by a thick area of coarse lamellar bone in which the blue areas represent the separation between the collagen bundles (zone 2). At the top right, a small area of immature osteoid (zone 3) is evident that is largely devoid of collagen. Electron microscopic findings in the femur biopsy (**g**) show abnormally curved and randomly oriented fibres of variable diameter that are easily identifiable within the matrix.

Plain radiographs showed a marked increase in bone density in a bilateral and symmetrical manner affecting the metaphyseal regions of the long bones including the distal femora (Figure 1a), proximal and distal tibiae, greater trochanteric region of the proximal femora, distal humeri, proximal radius, and ulnae. In the spine, marked osteopenia was evident with central collapse of the endplates that had a concave appearance (Figure 1b and c). A nuclear bone scan showed increased uptake in the bones around the knees and ankle joints in a symmetrical pattern at the sites of increased bone density. The bone mineral density of the lumbar spine and femoral neck was measured by dual X-ray absorptiometry (DXA) (Hologic Explorer). The spinal bone mineral density (BMD L1–4) was 0.532 g·cm^−2^ (T score −5.1) and the left femoral neck BMD was 0.631 g·cm^−2^ (T score −2.3). He received alendronate 70 mg per week, for 3–4 weeks.

A bone marrow biopsy and aspirate from the right posterior superior iliac spine carried out on the 10 April 2008 to further evaluate the significance of the circulating IgG kappa monoclonal protein showed that the marrow was normocellular with all cell lines present to maturity. There was a slight increase in megakaryocyte numbers. Very mild plasmacytosis was seen (3%) and kappa IgG was predominant. Chromosomal analysis was normal. A diagnosis of monoclonal gammopathy of uncertain significance was made. The bone biopsy obtained by trephine comprised cancelous bone and hemopoietic marrow. In some regions, sparse very slender trabeculae were noted and elsewhere the trabeculae had a thicker appearance. In the latter, widened osteoid seams were prevalent in which irregular calcification was noted giving the impression of prominent and wide cement lines. These were bordered by plump osteoblasts and admixed osteoclasts were present. On polarization microscopy, there was a paucity of collagen fibers within the thickened osteoid seams. In some of the thickened seams, residual spicules of original normal lamellar bone were identifiable (Figure 1d and e).Features suggesting microtrabecular fracture were also identified.

Congo red stain for amyloid was negative, granulomas were not noted, and mast cell numbers were unremarkable.

The radiological findings of axial osteopenia and appendicular sclerosis were considered highly unusual and the differential diagnoses considered included osteopetrosis, other sclerosing dysplasias, and osteogenesis imperfecta, all of which were considered unlikely given the presence of normal bone modeling. Sarcoidosis and mastocytosis (which can cause osteosclerosis) were considered unlikely given the bilateral symmetrical distribution of the abnormality and the bone marrow biopsy failed to support these. Fluorosis was considered unlikely as the abnormality was not confined to the axial skeleton and urinary fluoride was within normal limits. In juvenile Paget’s disease and hyperostosis in hyperphosphatemia, an earlier onset would be expected and involvement of the bone ends only would not be seen. Features of POEMS syndrome were excluded as there was no polyneuropathy, organomegaly, skin, or endocrine abnormalities, and myeloma, lymphoma, and myelofibrosis were excluded by bone marrow examination.

The main differential diagnoses ultimately included low-grade chronic osteomyelitis and a form of histiocytosis (Erdheim Chester disease specifically), although the symmetry of involvement made the former unlikely and the young age of the patient made the latter unlikely.

A subsequent open distal femoral biopsy to include cortex and medulla within the metaphyseal sclerotic area was performed on 14 June 2008 to further characterize the bony abnormality and to obtain bone for cell culture. At surgery the surgeon documented very hard bone. The biopsy comprised periosteum, cortex, and medulla measuring 30×10×4 mm and was suitable for morphometric, microbiologic, and electron microscopic examination. Cultures for aerobic and anaerobic bacteria were negative. Fungal elements were not seen. A stain for spirochetes was negative. Several fragments were placed in transport medium for cell culture. Despite the hardness of the bone documented at surgery, decalcification of the specimen was brisk, occurring within 4 h, suggesting ease of release of the mineral component. The component bony trabeculae within the medullary cavity were thick with a varying lamellar architecture. They were comprised of central zones of mature lamellar bone in which densely packed collagen with a smooth homogeneous polarization pattern was identifiable. This was bordered by broad zones of thick coarse lamellar bone in which the collagen fibers, although arranged in a lamellar pattern were distinctly separate one from one another. In some regions, these were in turn bordered by a third wave of ossification in which even less collagen content was demonstrable on polarization microscopy. These findings were confirmed with Masson Trichrome (Figure 1f) and reticulin staining. Scattered foci of osteoclast resorption were noted and in some regions resorption pits were filled with immature osteoid with diminished collagen content. Osteoblasts were inconspicuous throughout in contrast to the plump osteoblasts seen in the bone marrow biopsy.

The accompanying marrow was hemopoietic and somewhat hypocellular in nature. However, all cell lines were present to maturity. Plasma cells were identifiable with scattered areas in which increased numbers of plasma cells could be seen. These were polyclonal.

Electron microscopic examination showed seams of osteoid at the surface of the lamellar bone in which very sparse collagen fibrils of varying diameters were present. The collagen fibers were randomly oriented and many were small, irregularly shaped, and curved. Scattered irregular mineralization foci were present and mineral lay within a pale amorphous matrix (Figure 1g).This contrasts with normal bone where there is an orderly parallel orientation of collagen which is tightly arranged. The constellation of changes reflect those of diminishing collagen content within the osteoid of each modeling cycle accompanied by abnormal collagen fibers on electron microscopy. These features are consistent with those documented in fibrogenesis imperfecta ossium.^1–4^ Subsequently, from 2 months after the femoral bone biopsy, the patient received infusions of zolendronate, 4 mg, every 6 months. The spinal BMD showed a step-wise increase from 0.532 to 1.111 g·cm^−2^ (T score 0.2) over 7 years to 2015. In the same time interval, the left femoral neck BMD increased to 0.986 g·cm^−2^ (T score 0.4). In September 2009, the patient developed a fever and endocarditis due to salmonella. An aortic valve replacement was performed soon after.

In the interim, 10 000 units of vitamin D3 daily had been prescribed by an outside physician for a period of 2 years. Ultimately at 3 years after presentation to us, the serum 25OH vitamin D was 190 pmol·L^−1^ (NR 50–130) and the serum 1,25OH vitamin D was 370 pmol·L^−1^ (NR 38–160). At that time, the serum calcium was 2.32 mmol·L^−1^, normal. Thereafter, the dose of vitamin D was reduced to 1 000 units daily. Since that time, he has been pain free and fully mobile.

## Materials and methods

### Cell culture conditions

Osteoblasts were generated from bone biopsies obtained from the distal femur of the patient and from the distal femur of a male age 19 years (control) at the time of elective repair of an anterior cruciate ligament. Written informed consent was obtained from the patient and the study was approved by the Northern Sydney Local Health District Human Research and Ethics Committee. Cells derived from bone fragments were collected using trypsin (0.25%) and cultured in a 25 cm^2^ flask with high glucose Dulbecco’s modified Eagle medium and 10% (v/v) heat-inactivated fetal bovine serum, supplemented with penicillin (0.03 g·L^−1^) and streptomycin (0.04 g·L^−1^), and maintained at a 37 °C environment containing 5% (v/v) CO_2_.

In a separate experiment, the control osteoblasts were cultured with 10% normal serum or 10% patient’s serum instead of fetal bovine serum. In this latter culture system, the serum was not heat-inactivated.

### Biopsy cell morphology—scanning electron microscopy

Scanning electron microscopy was conducted to examine any morphological differences between the two biopsy-derived cells following long-term culture, as previously described by Slater *et al.*^5^ Cells were plated on Thermanox coverslips at a density of 1.25×10^5^ cells per well in six-well plates with culture medium and grown to confluence, with media changes every 3 days. Once cells were 30 days post confluence and had formed multilayers, cultures were fixed in 2.5% (w/v) glutaraldehyde in 0.1 mol·L^−1^ cacodylate buffer for 1 h at 4 °C, and washed three times with 0.1 mol·L^−1^ cacodylate buffer. Each coverslip was then post-fixed in 1%(w/v) osmium tetroxide in 0.1 mol·L^−1^ cacodylate buffer for 2 h at room temperature, washed three times with water, before dehydration with 30%, 50% and 70% (v/v) ethanol, three times each for 5 min. On the third wash, cells were left overnight in 70% (v/v) ethanol at 4 °C. Further dehydration with 90%, 95% and, finally, 100% (v/v) ethanol followed, each time for 10 min at room temperature. Cells underwent critical point drying, sputter-coated with 15 nm of gold palladium.

### Biopsy cell ultrastructural analysis—transmission electron microscopy

Transmission electron microscopy was employed to analyse the ultrastructural differences between the two biopsy-derived cells. Methodology was similar to that described above for scanning electron microscopy, with the following differences: cells were dehydrated using a graded series of ethanols as above, then placed in 25% and 50% (v/v) Spurr’s resin in ethanol for 1 h, followed by 2 h incubation in 75% (v/v) Spurr’s resin mixture. The multilayers were then left in 100% Spurr’s resin overnight at room temperature. At this stage, the coverslips were embedded in BEEM capsules and curing was carried out at 60 °C for 18 h.

### Biopsy cell proliferation assay (thymidine incorporation)

Various bone active agents were tested on both biopsy-derived cells to determine differences in cell proliferation and treatment responses. Cells were plated into 96-well plates at 1×10^5^ cells per well and incubated at 37 °C for 24 h. The medium was then replaced with serum-reduced OptiMEM media and further incubated for 24 h, before the addition of treatments (benzoylbenzoyl-ATP (BzATP) or 1,25-dihydroxyvitamin D in vehicle) for another 24 h. Tritiated thymidine (final concentration 20 nCi·μL^−1^) was added to each well and incubated for 4 h. Cell proliferation was indirectly determined by the incorporation of tritiated (methyl,1′2-^3^H) thymidine, as previously described.^6^ Scintillation vials were read for 5 min in a liquid beta-scintillation counter (Packard Tri-Carb 1990CA, Packard Instrument Company, Downers Grove, IL, USA). A bicinchoninic acid (BCA) assay was also carried out for cell proliferation measuring the amount of protein per well, in accordance with the manufacturer’s instructions.

### Osteoblast differentiation (alkaline phosphatase activity)

Cells were plated in 24-well plates and grown for 7 days, without additional treatments, to compare cell differentiation between the two biopsy-derived osteoblasts. Alkaline phosphatase was determined as previously described,^6^ using a modification of the method of Lowry^7^ that uses *p*-nitrophenyl phosphate as the substrate of alkaline phosphatase. Plates were read at 405 nm to detect the alkaline phosphatase product (*p*-nitrophenolate). Alkaline phosphate measurements were corrected for total cell protein using the BCA assay.

### RNA isolation and microarray analysis

DNA microarray technology was conducted to identify and classify a wide range of gene sequences expressed as messenger RNA (mRNA), to provide information on the differences in gene expression between the patient and control biopsy-derived cells. RNA was extracted from cells following 6 days in culture in six-well plates, using an RN easy Mini kit (Qiagen, Hilden, Germany) according to the manufacturer’s instructions. In a second experiment, RNA was extracted from the control osteoblasts after culture with normal serum or the patient’s serum for 6 days. Quality control was performed to determine microarray suitability. RNA yield and integrity was evaluated by both NanoDrop spectrophotometry (Nanodrop Technologies, Wilmington, DE, USA) and 1%(w/v) agarose electrophoresis. Pure quality RNA samples were then dispatched to the Ramaciotti Centre at the University of New South Wales for a full service Affymetrix Gene Array analysis. Results were downloaded to the microarray program, GeneSpring GX. In the second experiment involving culture of control osteoblasts with either normal serum or the patient’s serum, the Affymetrix gene microarray analysis was carried out by the Department of Bioinformatics, University of Queensland (QFAB).

### Tumor necrosis factor receptor 11B (OPG)

Secreted osteoprotegerin (OPG) protein expression was examined for both the control and patient biopsy-derived cells. Cells were plated in 24-well plates in quadruplicates and grown for 7 days. Supernatant at both 4 and 7 days in culture were collected from each well and tested for OPG levels using an ELISA assay.^8^ The Biomedica enzyme-linked immunosorbent assay (ELISA) test kits were enzyme immunoassays designed to determine OPG directly in cell culture supernates. Cell protein corrections were made using a BCA assay of cell lysates from the original plates.

### Sclerostin and vitamin D receptor (western blot)

Cells were plated in 24-well plates and cultured for 20 days post confluence for sclerostin and for 7 days for the vitamin D receptor (VDR). The attached cells were washed once with ice-cold phosphate-buffered saline (PBS) buffer before being lysed in ice-cold RIPA buffer (50 nmol·L^−1^ Tris,150 mmol·L^−1^ NaCl, 1 mmol·L^−1^ EDTA, 1% NP40+ protease inhibitors (1 mmol·L^−1^ PMSF, 1 μmol·L^−1^ Becstatin, HCl, 1 μmol·L^−1^ Pepstatin A) for 5 min.The cell lysate was stored at −20 °C until needed for western blot analysis, as previously described.^8^ The protein concentrations were determined by BCA assay and equal amounts of protein were subject to electrophoresis on 10% SDS-polyacrylamide gel electrophoresis for sclerostin and VDR and then transferred electrophoretically onto a nitrocellulose membrane and incubated in 1% (w/v) heat-denatured casein in PBS containing 0.04% (w/v) thymol (HDC) for 1 h at room temperature. Membranes were then exposed to primary antibody (1 μg·mL^−1^ anti-human sclerostin and 1 μg·mL^−1^ anti-human VDR) in HDC for 1 h at room temperature, washed several times with 0.05% (v/v) Tween-20 in PBS, and incubated with secondary anti-IgG horse radish peroxidase-conjugated antibody for 1 h at room temperature. Membranes were washed extensively and an enhanced chemiluminescence detection assay (Millipore, Millipore Corporation, Cork, Ireland) was performed according to the manufacturer’s instructions. Band densities were measured using an Alpha Innotech Digital Imaging System (Genetic Technologies Inc, Miami, FL, USA).

## Results

Osteoblasts obtained from the patient’s bone biopsy were established in culture. Osteoblasts obtained from a bone biopsy of the lower femur of a healthy 19-year-old male having elective knee surgery for a torn anterior cruciate ligament were used as a control.

### Morphological and ultrastructural differences in biopsy cells following long-term culture

**Figure 2:**
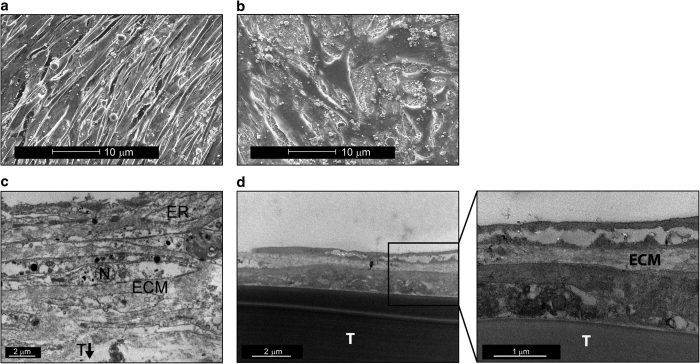
Scanning electron microscopy of control osteoblasts (**a**) cultured for 30 days post confluence. Scanning electron microscopy of the patient’s osteoblasts (**b**) cultured for 30 days post confluence. Transmission electron microscopy of control osteoblasts (**c**) cultured for 30 days post confluence (ECM, extracellcular collagen matrix; ER, endoplasmic reticulum; N, osteoblast cell nucleus; T, thermanox coverslips). Transmission electronic microscopy of the patient’s osteoblasts (**d**) cultured for 30 days post confluence.

Long-term culture of osteoblasts results in the formation of multilayers on Thermanox consisting of a collagen-based extracellular matrix, presented in an organized manner. This phenomenon has been shown previously *in vitro* using fetal bone cells, with multilayers becoming macroscopic at ~20 days post confluence.^9^ Scanning electron microscopy was conducted to give a more detailed view of the biopsy-derived osteoblasts (control and patient) and the morphologic differences of the surface layer after long-term culture. The control cells exhibited an elongated uniform structure very similar to fetal bone cells (Figure 2a). In contrast, the patient cells appeared very flat and disorganized, very different to any known or previously documented osteoblast (Figure 2b).These SEM images provide evidence of phenotypic differences between the two biopsy-derived cells in culture.

Transmission electron microscopy was employed to examine the multilayering of the biopsy-derived osteoblasts (control and patient) in finer detail. This was to investigate the ultrastructural organization of the collagen layering between these cells following long-term culture, using fetal bone cells as a positive control. The control biopsy-derived osteoblasts showed similar multilayers to those observed with fetal bone cells (Figure 2c). The patient-derived cells appeared quite disorganized and without any well-defined features, such as multiple cell layers and endoplasmic reticulum, both of which are seen in the other cell types (Figure 2d).

### Cell proliferation is reduced in the patient biopsy-derived osteoblast cells

**Figure 3:**
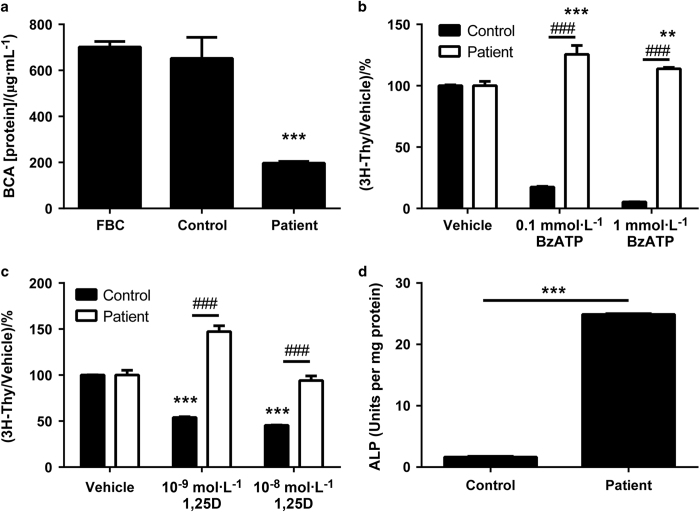
Protein production by cultured osteoblasts (**a**). FBC, fetal bone cells. ***P<0.001 compared with control. 3H thymidine incorporation into osteoblasts in culture BzATP (**b**). ***P<0.001 compared with vehicle; **P<0.01 compared with vehicle; ^###^*P*<0.001 compared with control. 3H thymidine incorporation into osteoblasts in culture (**c**). 1,25D, 1,25-dihydroxyvitamin D. ***P<0.001 compared with vehicle; ^###^*P*<0.001 compared with control. Alkaline phosphatase activity in cultured osteoblasts (**d**). ***P<0.001 compared with control.

The growth of the patient-derived osteoblasts appeared much slower than the control cells under the specified culture conditions. Therefore, assays for protein (BCA) production by the cells and thymidine incorporation into the cells were performed to quantify these apparent cell replication differences. Cell proliferation between fetal bone cells, control, and patient cells were all compared by protein measurement, following 5 days in culture without any treatment and also by the measurement of thymidine incorporation into the cells. Proliferation of patient cells was significantly lower in comparison with control biopsy cells and fetal bone cells. (Figure 3a).

### Effects of bone active agents on the proliferation of biopsy-derived osteoblasts

Both the patient and control osteoblasts expressed the P2X7 receptor and the VDR.

The proliferation rate of the control biopsy-derived cells significantly decreased as BzATP concentrations increased (*P*<0.001). In contrast, the patient biopsy-derived cells showed increased proliferation in response to BzATP, an agonist of the BzATP receptor, with significant increases at both the lower 0.1 mmol·L^−1^ BzATP dose (*P*<0.001) and the higher 1 mmol·L^−1^ BzATP dose (*P*<0.01; Figure 3b).

Similar to the effects seen with BzATP, the patient’s cells responded in a paradoxical manner when treated with 1,25-dihydroxyvitamin D. Usually with increasing concentrations of 1,25-dihydroxyvitamin D, osteoblast cell proliferation decreases^6,10^ and this was seen in the control biopsy cells. The patient cells, however, significantly increased proliferation in culture with a concentration of 10^−9^ mol·L^−1^ 1,25-dihydroxyvitamin D concentration(*P*<0.001; Figure 3c).

Alkaline phosphatase was used as a marker of osteoblastic differentiation for control and patient biopsy-derived bone cells, and was corrected for total cellular protein as measured by the BCA assay. These cells, similar to fetal bone cells, had a maximal alkaline phosphatase level after 7 days in culture. A comparison was made between the two biopsy osteoblasts, with no additional treatments. A significant increase in alkaline phosphatase activity was evident in the patient’s osteoblasts (*P*<0.001) in comparison with the control biopsy osteoblasts, a difference that reached 10-fold (Figure 3d).

### Affymetrix gene array

#### Gene microarray analysis comparing the patient’s osteoblasts with osteoblasts from a normal control in culture

A microarray was performed on mRNA extracted from the control and patient-derived osteoblasts after 6 days in culture to compare differences in gene expression levels between the two cell types. The mRNA underwent quality control before microarray analysis. The genes showing the most significant upregulation and downregulation are shown in Table 2. The most upregulated gene in the patient’s osteoblasts compared with the control osteoblasts was G-protein-coupled receptor 128, an orphan receptor of unknown function. The most downregulated gene was chemokine, cc motif, receptor-like protein 1 (CCRL1), of unknown relevance to bone cell function. RANK expression in osteoclasts is upregulated by chemokine receptor 2 (CCR2).^11^ Tumor necrosis factor receptor 11b (OPG) gene expression was markedly upregulated. Secreted OPG protein was measured in both the control and patient biopsy-derived cells by ELISA. All OPG protein levels measured were corrected for total protein content, as assessed by BCA assay. OPG protein expression was increased significantly (*P*<0.001) in the patient cells compared with control cells, confirming the microarray results for mRNA (Supplementary Figure 1). This increase resulted in a 10-fold difference. Because of the possibility that the diminished collagen observed in cell culture experiments might be due to reduced synthesis of collagen, the expression of collagen genes was examined.COL1A1, COL1A2, COL3A1, and COL3A2 were not significantly upregulated or downregulated. COL4A1, COL4A2, and COL11A1 were significantly upregulated, COL11A1 3.25-fold. Collagen protein synthesis was not measured. An immunostain of bone was negative for collagen 4.

The expression of the sclerostin (SOST) protein was examined in control and patient biopsy-derived cells maintained in culture, by western blot analysis to test the microarray analysis which showed no significant fold change. The expression of sclerostin protein examined by this technique did not show any significant difference between the control and the patient biopsy-derived cells.(Supplementary Figure 2).

The expression of VDR gene was upregulated 2.58-fold. The VDR protein was also measured in control and patient biopsy-derived cells, maintained in culture, by western blot analysis of protein expression, and no significant difference between the cell types was seen (Supplementary Figure 3).

#### Gene microarray analysis comparing control osteoblasts cultured with the patient’s serum vs the same normal osteoblasts cultured with control serum

The control serum was taken from a different healthy young male patient. The control serum and the patient’s serum, after centrifugation to remove red cells and white cells, were added in equal amounts to separate incubation medium to comprise 10% of the incubation medium. The testosterone concentration was measured by Liquid Chromatography-Mass Spectrometry (LC-MS) of the incubation medium prepared with the patient’s serum and with the control serum, and the values were 1.14 nmol·L^−1^ and 1.60 nmol·L^−1^, respectively.

A microarray was performed on mRNA extracted from the control osteoblast after 6 days in culture where the culture had been performed with the patient’s serum or control serum. The genes showing the most significant upregulation and downregulation are shown in Table 3. The gene expression seen in control osteoblasts cultured with the patient’s serum was quite different from the gene expression seen when the control osteoblasts were cultured in normal media. The most upregulated gene was aquaporin 1 (AQP1) and the most downregulated gene was metallothionein (MT1G). The following genes which might be relevant to bone cell metabolism were neither significantly upregulated or downregulated: ectonucleotide pyrophosphatase 1 (ENPP1), osteopontin (SPP1), osteocalcin (GLAP), bone sialoprotein (IBSP), matrix extracellular matrix phosphoglycoprotein (MEPE), dentin matrix acidic phosphoprotein (DMP1), complement component 1, q1-subcompartment A and B chains (C1QA, C1QB). Selected genes were further studied by reverse transcription PCR. The data showed significant upregulation of AQP1 after 6 days of culture. A significant increase in cartilage intermediate layer protein (CILP) was confirmed. Significant downregulation of serpin peptidase inhibitor clade A (SERPINA1) and secreted frizzled-related protein 4 (SRP4) were seen. No significant changes in C1QA or C1QB were seen.

## Discussion

In 1950, Baker and Turnbull^12^ described two patients in whom features resembling osteomalacia were present that pursued a relentlessly progressive course. The disorder, initially referred to as “Baker’s disease”, was subsequently named fibrogenesis imperfecta ossium reflecting abnormal bone matrix lacking normal lamellar bifringence of collagen with polarized light and associated with a large amount of incompletely calcified osteoid. The histopathological findings in the disorder were initially described in 1956 by Baker^13^ and subsequently expanded on by many authors.^1–4,14,15^ All cases showed similar histological findings of varying degree of severity. Thickened trabeculae with pale wide osteoid seams were documented in which diminished or deficient collagen was associated with coarse granular calcification. Bone collagen only was affected and variable quantities of residual lamellar bone were identified depending on the stage of evolution. The calcified zones abut normal trabeculae with diminishing calcification in the outer zones and variable quantities of residual host lamellar bone. This entity differs from osteomalacia in that in osteomalacia the collagen content is normal. The abnormality spares periosteum, ligaments, tendons, and fracture callus. The changes in the cortex were less developed than in the medulla^15^ and this was seen also in our case.

Electron microscopic findings showed that the numbers of collagen fibers are diminished, have a random orientation, and are commonly curved and irregular with a variable diameter of 22–136 nm. Commonly on electron microscopy random scattered calcified nodules were present. These were increased in the area near the calcified bone and decreased in the sparsely collagenized zones. Amorphous matrix predominated in the non-collagenous area. Osteoblasts, osteocytes, and osteoclasts have not previously been found to be abnormal.^1–4,14,15^ The abnormal trabecular pattern of bone was noted on imaging by Golding in 1968.^16^ Several authors have emphasized an altering appearance on imaging over time.^4,17^ Collagen is required for mineralization. In the presence of residual collagen, the mineral content was found to be increased.^13,15^ These authors suggested that the less collagen content initially allowed increased space for mineralization explaining the sclerosis seen on X-ray and the fact that it was extremely hard at biopsy. However, the mineral is poorly bound with ease of decalcification as exemplified in our case where there was easy rapid decalcification. The sclerosis reduces with diminution of collagen over time reflecting a lack of calcium binding.

Baker *et al* in 1966^18^ described a third case emphasizing the abnormally coarse trabecular pattern of bone with increasing bone density particularly in the metaphyseal portion of long bones. In time, further cases were documented^19^ and over the years it appears that of the order of 25–30 cases only have been described. An inhibitor of matrix calcification in the serum was not found^20^ and the serum pyrophosphate was normal. Henneman *et al*^21^ found that the soluble component of collagen, that is newly synthesized collagen, was increased but total mature collagen was reduced. It had been previously reported that cross-linking of collagen was associated with the mineralization process.^22^ Stoddart *et al*^23^ had documented a case with a circulating paraprotein, subsequently confirmed in several further cases in which a circulating paraprotein usually an IgG kappa or IgG lamda was found^3,17,24–26^ Patients with the disorder have been treated with melphalan and prednisolone with histological improvement,^4,24^ but others have not shown benefit with melphalan.^25^ Bakos *et al*^26^ described a patient whose severe diffuse skeletal pain was reduced by plasmapharesis.

In our case, the serum alkaline phosphatase was elevated, 206 U·L^−1^, a consistent finding in other cases, with some values over 500 U·L^−1^.^4,26^ The serum levels of C3 and C4 were persistently diminished, a hitherto undocumented finding. There was no evidence of other disorders in which this may occur, including systemic lupus erythematosus and glomerulonephritis. The low level of C3 may have increased his susceptibility to the salmonella bacterial infection that damaged his aortic valve. Presumably the deficiency of C3 and C4 were acquired as there was no history of recurrent infections during childhood. Increased susceptibility to both Gram-positive and Gram-negative bacterial infection have been described with C3 deficiency.^27,28^ Osteoblasts can be stimulated to produce C3 by 1,25-dihydroxyvitamin D.^29^ In culture experiments when anti-C3 antibody was used, osteoclast formation was greatly inhibited.^30^There has been no evidence for cleavage of C3 in either osteoblasts or osteoclasts.^31^ C1-s can cleave type I and type II collagen^32^ but the possible role of any component of the complement system in the mineralization of collagen is unknown. In fibrogenesis imperfecta ossium, there appears to be a defect in the cross-linking of collagen and subsequent failure to appropriately calcify the collagen matrix. A somewhat similar histologic pattern has been observed in osteogenesis imperfecta type VI.^33^However, in that entity polarizable collagen is present, albeit in an abnormal distribution giving a so called “fish scale” appearance. A complete lack of collagen with diminution in quantity over time as seen in fibrogenesis imperfecta ossium (FIO) is not documented. The genetic mutation in osteogenesis imperfecta type VI has been identified as a homozygous mutation in SERPIN F1.

In our case, the combination of clinically diffuse bone pain, imaging findings of severe osteopenia in the axial skeleton, increased metaphyseal bone density in the long bones, and bone biopsy at both sites in which abnormal bone remodeling with increased osteoid seams comprising diminished collagen identifiable with polarized light, fits the criteria for a diagnosis of fibrogenesis imperfecta ossium. A new finding, hitherto undocumented, of a low serum C3 and C4 in combination with a circulating IgG kappa paraprotein, a finding noted in prior cases, was identified, the significance of which to the pathogenesis of the disorder is unknown. Additional biochemical abnormalities were a significantly elevated serum TRACP-5B reflecting increased osteoclast activity, a feature not overtly identified in bone biopsy specimens, a persistently elevated serum inorganic phosphate, and an abnormal increase in 1,25-dihydroxyvitamin D after treatment with vitamin D. The serum calcium and serum phosphate have been consistently normal in other cases.

In an attempt to gain further insights into the pathogenesis of the bone disorder, osteoblasts were successfully derived and cultured from a biopsy obtained from the distal femur and compared with osteoblasts derived and cultured from the distal femur of a 19-year-old patient undergoing elective knee surgery deemed to be a normal control. The selected control was a healthy male close in age to our patient. Both had suffered a rupture of the anterior cruciate ligament. In both cases the biopsy was obtained from the lower femur. The osteoblasts obtained from the control biopsy behaved in culture in a remarkably similar manner to fetal bone cells (primary human osteoblasts),^9^ and were markedly different in behavior to the osteoblasts obtained from the patient. This has led us to believe that the “control” osteoblasts in culture could be used as an appropriate control to compare with the patient’s osteoblasts. In culture, the osteoblasts obtained from the patient showed marked morphological differences when compared with osteoblasts grown in culture from the control. The rate of proliferation of the patient’s osteoblasts was much less than that of the control osteoblasts. Alkaline phosphatase activity was much higher in the patient’s osteoblasts compared with controls. Paradoxically in contrast to the response in the control osteoblasts, 1,25-dihydroxyvitamin D markedly stimulated proliferation of the patient’s osteoblasts.

In a gene microarray study carried out on mRNA extracted from the patient’s osteoblasts and the control osteoblasts after 6 days in culture, OPG mRNA expression was markedly increased in the patient’s osteoblasts when compared with the control osteoblasts and OPG protein measured by ELISA was also markedly increased in the patient’s osteoblasts. This would be expected to diminish osteoclast activity but in fact the serum TRACP-5B was increased reflecting increased osteoclast activity. The expression of the VDR protein examined by western blot was not increased in the patient’s osteoblasts, although the cells increased their rate of proliferation when cultured with 1,25-dihydroxyvitamin D.

Several other genes that might be involved in bone cell function were either markedly upregulated or downregulated in the gene microarray study. Osteopontin, upregulated, inhibits hydroxyapatite formation after phosphorylation. Alkaline phosphatase dephosphorylates osteopontin. Pyrophosphate upregulates osteopontin, and pyrophosphate and osteopontin together inhibit bone mineralization.^34^CD36 (collagen type 1 receptor), upregulated in the patient’s osteoblasts, could be involved in the genesis of osteoporosis through stimulation of bone resorption.^35^ The upregulation of collagen type IV alpha 1 and alpha 2 subunits has been linked to low bone mass.^36^ Tumor necrosis factor receptor 11B (OPG) mRNA and protein expression were upregulated and by binding to RANKL could lead to unrestrained bone formation.^37^ Secreted frizzled-related protein 2 (SFRP2) was upregulated. The expression of SFRP2 is strongly upregulated during osteoblastic differentiation^38^ and inhibits Wnt3a/beta catenin signaling.^39^ Cartilage oligomeric matrix protein (COMP), upregulated, has been implicated in the development of irregular ossification in epiphyses,^40^ and in the development of abnormal collagen fibril morphology.^41^ SFRP1 was downregulated. SFRP1^−/−^ knockout was associated with increased osteoblastic proliferation and differentiation, and increased trabecular bone formation.^42^ T-box 3 is a key promoter of osteoblast proliferation^43^ and was downregulated in the present study. The bone biopsy was taken from an area in the femur of increased bone density and the increased expression of alkaline phosphatase and the paradoxical response to 1,25-didroxyvitamin D of the osteoblasts in culture suggest that factors facilitating bone formation were dominant.

In the study in which control osteoblastic cells in culture were exposed to the patient’s serum AQP1 was the most upregulated mRNA. The function of this gene in osteoblasts is unknown but is highly expressed in osteosarcoma cells. Knockdown of AQP1 was associated with reduced proliferation of osteosarcoma cells.^44^ R-spondin-3 was downregulated. R-spondins may enhance Wnt/beta catenin signaling.^45^ SFRP4 was downregulated. SFRP4 has a phosphaturic action similar to that of FGF-23,^46^ and its downregulation may be relevant to the persistent hyperphosphatemia seen in this patient. In the patient’s poorly proliferating cells bone morphogenetic protein 2 (BMP2) was downregulated. BMP2 induces bone formation and osteoblastic differentiation in association with Runx2 and osterix expression.^47^ BMP2 expression is activated by Wnt14 and Wnt14, and BMP2 synergize to promote osteoblastic differentiation.^48^ Neither overexpression nor underexpression of any of the Wnt ligands was noted in the present study. Cross-linking of collagen is important for hydroxyapatite deposition on collagen fibrils. Lysyl hydroxylase is important in the cross-linking process.^49^ Inhibition of lysyl hydroxylase gives irregularly shaped collagen fibrils. Lysyl oxidase is also involved in cross-linking of collagen and is markedly upregulated in the differentiation of osteoblasts.^50^ The genes coding these enzymes were neither upregulated nor downregulated in the present study. Collagen alone cannot initiate hydroxyapatite nucleation on collagen fibrils.^51^ Bone sialoprotein (BSP) that is expressed in and secreted by osteoblasts is necessary to initiate hydroxyapatite deposition.^52^ BSP binds to collagen1alpha 2 to stimulate matrix calcification.^53^ BSP mRNA was neither upregulated nor downregulated in the present study. Another gene upregulated to a lesser extent was ROR2 (4.63-fold), which is a receptor for Wnt5a and activates the non-canonical pathway in osteoblasts. Overexpression of ROR2 increased osteoblastic differentiation^54^ and this mechanism may be relevant in our patient with increased bone mass at the site of biopsy. Given that three to four tablets of alendronate were taken before the femoral bone biopsy, the possibility that these significantly influenced the gene expression in osteoblasts should be considered. Gene expression in osteoblasts has been studied in rhesus monkeys treated with alendronate.^55^ The OPG gene was upregulated 16-fold compared with 17.26 upregulation in our study. ROR2 was downregulated in the rhesus monkey study but upregulated in our study in which the patient’s osteoblasts were compared with control osteoblasts in culture.SPP1 (osteopontin) was downregulated 25-fold in the rhesus monkey osteoblasts compared with a 32.06 upregulation in our study. When control osteoblasts were cultured in the patient’s serum SFRP4 was downregulated, a similar finding to that in rhesus monkey osteoblasts. None of the other genes listed in Tables 2 and 3 were significantly upregulated or downregulated in the rhesus monkey study.

In summary, this patient had wide spread bone pain, reduced vertebral bone density, marked sclerosis at the end of long bones, and a bone biopsy that showed diminished collagen content on light and electron microscopy, with reduced collagen birefringence on polarized light microscopy consistent with the diagnosis of fibrogenesis imperfecta ossium. A circulating IgG kappa paraprotein was identified and there was marked reduction in serum C3 and C4 a finding not previously reported in this disorder. More information about the possible role of C3 and C4 in the proliferation, differentiation and function of osteoblasts is required. Osteoblasts cultured from the femoral bone biopsy obtained from the patient showed abnormal morphology, reduced proliferation rates and responded abnormally to 1,25-dihydroxyvitamin D. A gene expression study performed on mRNA obtained from the osteoblasts showed marked upregulation of GPR 128 an orphan receptor of unknown function. Control osteoblasts in culture obtained from a normal male of similar age showed increased expression of AQP1 when cultured with serum from the patient. These are the first studies to examine proliferation, protein production and gene expression in osteoblasts in this disorder. A single factor defining the pathogenesis of this disorder has not been discovered. A successful treatment for fibrogenesis imperfecta ossium has not been forthcoming. Treatment with melphalan has not been consistently effective. The use of bortezomib was considered but not pursued. The use of plasmapharesis has been described in one case. In the present case, the use of zolendronate infusions initially every 6 months has resulted in a marked increase in spinal BMD and a marked reduction in joint and muscle pain.

## Figures and Tables

**Table 1:** Investigations

Test	Results	NR
Hemoglobin	119 g·L^−1^	130–160
White cell count	Normal	4–11
ESR	45 mm·h^−1^	<15
Hepatitis B surface antigen	Negative	—
Hepatitis C antibody	Negative	—
Serum C3	0.38 g·L^−1^	0.83–1.46
Serum C4	0.04 g·L^−1^	0.16–0.45
TreponemaAb	Negative	—
ASOT	345	<300
ANA	Negative	—
HIV Ab	Negative	—
TSH	2.05 mU·L^−1^	0.4–4.0
BorreliaAbIgG, IgM	Negative	—
Serum creatinine	70 μm·L^−1^	60–100
Serum calcium	2.34 mmol·L^−1^	2.20–2.55
Serum alkaline phosphatase	206 U·L^−1^	41–119
Serum phosphate	2.14 mmol·L^−1^	0.78–1.43
Serum 25OH Vitamin D	49.8 nmol·L^−1^	33.1–129
Serum 1,25OH Vitamin D	109 pmol·L^−1^	38–160
Serum PTH	29.8 ng·L^−1^	<50.0
24-hour urine calcium	7.29 mmol	1.25–7.50
Serum testosterone	15.5 nmol·L^−1^	8.5–55.5
Urine deoxypyridinoline	11.4 nmol per mmol Cr	—
TRACP-5ß	10.1 U·L^−1^	<4.8
Serum calcitonin	<5 ng·L^−1^	—
Serum ferritin	167 μg·L^−1^	30–400
CRP	44 mg·L^−1^	<5
Rheumatoid factor	<20 IU·mL^−1^	—
Serum osteocalcin	4.9 μg·L^−1^	3.7–10.0
Q fever and rickettsial antibodies	Negative	—
Leptospira IgM	Negative	—
IgG kappa monoclonal protein	3.9 g·L^−1^	—
Serum-free kappa light chains	26.3 mg·L^−1^	3.3–19.4
Anti-transglutaminaseAb	<3 U·mL^−1^	—
Serum acid phosphatase	10.1 U·L^−1^	<6.6
16srRNA	Not detected	—
Serum vitamin A	2.2 μmol·L^−1^	0.7–3.0
Blood lead, cadmium, and mercury	Negative	—
Urine fluoride	4.60 mmol per mol Cr	Biological occupational exposure limit 42

**Table 2:** The fold change in gene expression

Gene status	Gene name	Gene symbol	Fold change
Genes upregulated	G-protein-coupled receptor 128	GPR 128	56.24
	Ankyrin repeat-domain-containing protein 1	ANKRD1	37.53
	EGF containing fibulin-like extracellular matrix 1	EFEMP1	36.28
	Secreted phosphoprotein 1 (osteopontin)	SPP1	32.06
	CD36 (collagen type 1 receptor)	CD36	30.59
	Vascular cell adhesion molecule 1	VCAM 1	21.68
	Cadherin 13	CDH 13	18.53
	Flavin containing mono-oxygenase 3	FMO3	18.51
	Collagen type IV alpha 1	COL4A1	17.39
	Tumour necrosis factor receptor 11B	TNRSF11B	17.26
	Lipid phosphate phosphatase related 4	LPPR4	14.18
	Secreted frizzled-related protein 2	SFRP2	13.22
	Collagen type IV alpha 2	COL4A2	9.25
	Cartilage oligomeric matrix protein	COMP	8.09
	Vitamin D receptor	VDR	2.58
Genes downregulated	Chemokine, cc motif, receptor-like protein	CCRL1	31.19
	Carboxypeptidase X, M_2_	CPXM2	19.50
	Prostaglandin 12 synthase	PTG1S	14.48
	Zinc-finger protein FOG family member 2	ZFPM2	13.55
	T-Box5	TBX5	10.57
	Angiomotin	AMOT	9.72
	Calmegin	CLGN	9.41
	Scavenger receptor class A member 3	SCARA3	9.37
	Secreted frizzled-related protein 1	SFRP1	6.05
	T-Box3	TBX3	5.33

Patient’s osteoblasts in culture compared with control osteoblasts after 6 days in culture.

**Table 3:** The fold change in gene expression

Gene status	Gene name	Gene symbol	Fold change
Genes upregulated	Aquaporin 1	AQP1	8.28
	Cartilage intermediate layer protein	CILP	7.47
	Calsequestrin 2	CASQ2	7.21
	Retinol-binding protein 1	RBP1	7.11
	Nephronectin	NPNT	6.84
	Myosin light-chain alkali	MYL 1	6.22
	SRY-box 11	SOX 11	6.15
	Troponin C type 2	TNNC2	5.71
	Breast carcinoma amplifier sequence 1	BCAS1	5.32
	Heat shock 27 kDa protein 3	HSPB3	5.21
Genes downregulated	Metallothionein	MT1G	7.27
	Oligodendrocyte myelin glycoprotein	OMG	5.67
	Serpin peptidase inhibitor clade A	SERPIN A1	4.67
	Matrix metallopeptidase 3	MMP3	4.65
	Prostaglandin F receptor	PTGFR	4.38
	Fbj murine osteosarcoma viral oncogene	FOS	3.82
	R-spondin-3	RSPO3	3.71
	Aquaporin 9	AQP9	3.43
	Proteoglycan 4	PRG4	3.20
	Suprabasin	SBSN	3.05
	Secreted frizzled-related protein 4	SFRP4	2.68
	Bone morphogenetic protein 2	BMP2	2.15

Control osteoblasts cultured in patient’s serum compared with control osteoblasts cultured in normal serum after 6 days in culture.
