# Supplementary material for: Clinical, cellular, microscopic, and ultrastructural studies of a case of fibrogenesis imperfecta ossium
Source: Bone Res. 2017 Mar 14;5:16057–. doi: 10.1038/boneres.2016.57 (PMC5350113; doi:10.1038/boneres.2016.57)
Supplement: Supplementary Figures [file boneres201657-s1.doc]

**SUPPLEMENTARY FIGURES**


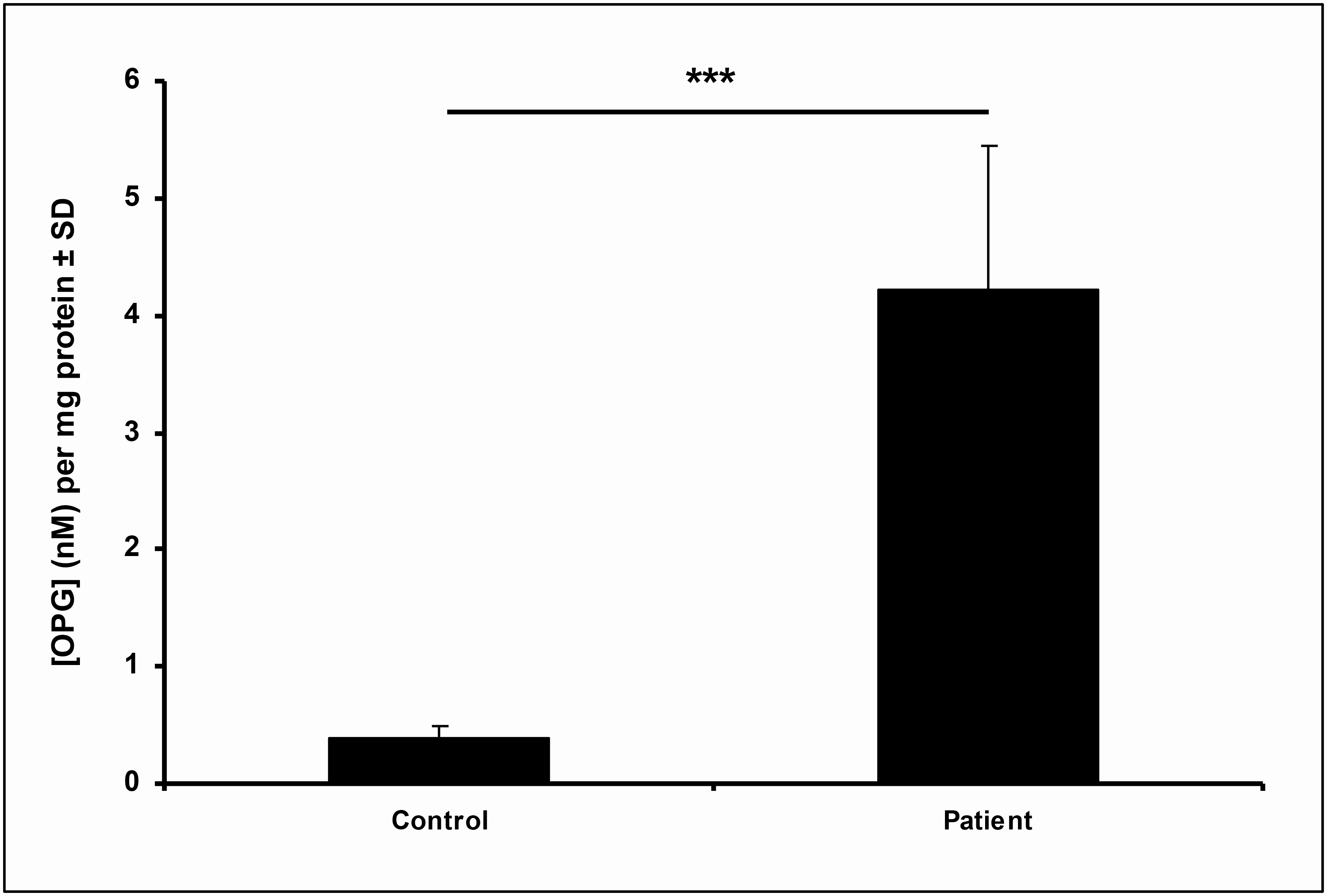


**Supplementary Figure 1:** Cells were plated in 24-well plates and grown in quadruplicates for 7 days. The supernatant from each well was measured for OPG with an ELISA and corrected for total cell protein using BCA assay. Results are presented as means ± standard deviations from 2 independent experiments. Significantly different: *** p < 0.001.


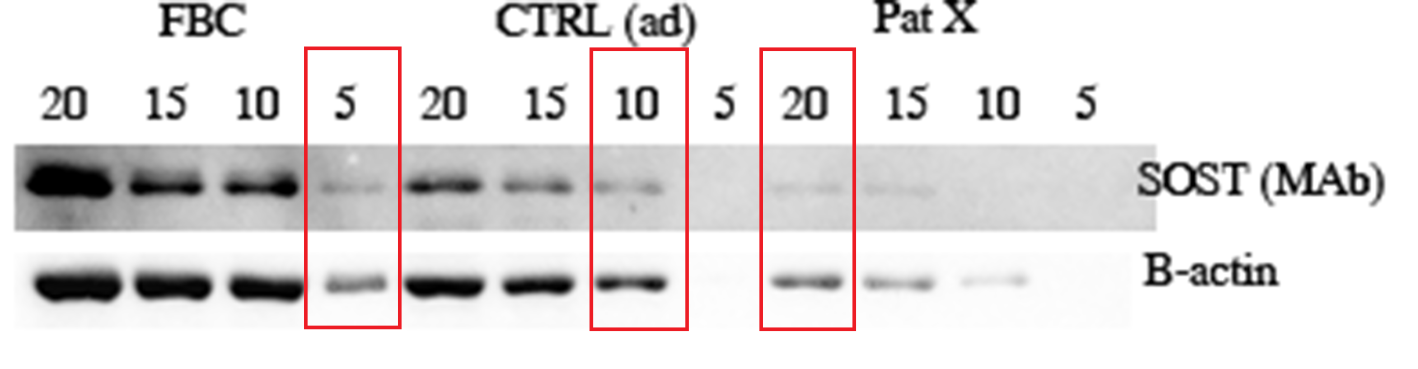


**Supplementary Figure 2:** All cells types were plates in 24-well plates and grown in quadruplicates for 20 days post confluence. Cells were lysed in RIPA buffer and sclerostin protein expression was measured by western blot analysis. β-actin was used as the loading control. Boxes indicate total amount of protein loaded for each sample that equated to roughly equivalent amounts of β-actin.


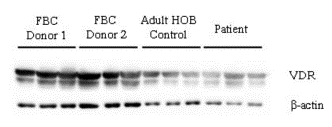


**Supplementary Figure 3:** All cell types were plates in 24-well plates in triplicates for 7 days. Cells were lysed in RIPA buffer and vitamin D receptor expression was measured by western blot analysis. Β-actin was used as the loading control.
